# A Perfusion Bioreactor System for Cell Seeding and Oxygen-Controlled Cultivation of Three-Dimensional Cell Cultures

**DOI:** 10.1089/ten.tec.2018.0204

**Published:** 2018-10-17

**Authors:** Jakob Schmid, Sascha Schwarz, Robert Meier-Staude, Stefanie Sudhop, Hauke Clausen-Schaumann, Matthias Schieker, Robert Huber

**Affiliations:** ^1^ Center for Applied Tissue Engineering and Regenerative Medicine (CANTER), University of Applied Sciences Munich, Munich, Germany.; ^2^ Laboratory of Experimental Surgery and Regenerative Medicine (ExperiMed), Ludwig-Maximilians University Munich, Munich, Germany.; ^3^ Department of Industrial Engineering and Management, University of Applied Sciences Munich, Munich, Germany.; ^4^ Department of Mechanical Engineering, Technical University Munich, Garching, Germany.; ^5^ Center for Nanoscience (CeNS), Ludwig-Maximilians University Munich, Munich, Germany.

**Keywords:** oxygen measurement, feedback control, cell seeding, perfusion microbioreactor, 3D cell culture

## Abstract

Bioreactor systems facilitate three-dimensional (3D) cell culture by coping with limitations of static cultivation techniques. To allow for the investigation of proper cultivation conditions and the reproducible generation of tissue-engineered grafts, a bioreactor system, which comprises the control of crucial cultivation parameters in independent-operating parallel bioreactors, is beneficial. Furthermore, the use of a bioreactor as an automated cell seeding tool enables even cell distributions on stable scaffolds. In this study, we developed a perfusion microbioreactor system, which enables the cultivation of 3D cell cultures in an oxygen-controlled environment in up to four independent-operating bioreactors. Therefore, perfusion microbioreactors were designed with the help of computer-aided design, and manufactured using the 3D printing technologies stereolithography and fused deposition modeling. A uniform flow distribution in the microbioreactor was shown using a computational fluid dynamics model. For oxygen measurements, microsensors were integrated in the bioreactors to measure the oxygen concentration (OC) in the geometric center of the 3D cell cultures. To control the OC in each bioreactor independently, an automated feedback loop was developed, which adjusts the perfusion velocity according to the oxygen sensor signal. Furthermore, an automated cell seeding protocol was implemented to facilitate the even distribution of cells within a stable scaffold in a reproducible way. As proof of concept, the human mesenchymal stem cell line SCP-1 was seeded on bovine cancellous bone matrix of 1 cm^3^ and cultivated in the developed microbioreactor system at different oxygen levels. The oxygen control was capable to maintain preset oxygen levels ±0.5% over a cultivation period of several days. Using the automated cell seeding procedure resulted in evenly distributed cells within a stable scaffold. In summary, the developed microbioreactor system enables the cultivation of 3D cell cultures in an automated and thus reproducible way by providing up to four independently operating, oxygen-controlled bioreactors. In combination with the automated cell seeding procedure, the bioreactor system opens up new possibilities to conduct more reproducible experiments to investigate optimal cultivation parameters and to generate tissue-engineering grafts in an oxygen-controlled environment.

## Impact Statement

The article describes a novel parallelized perfusion microbioreactor system, which is manufactured using three-dimensional printing technology. The microbioreactor system enables the cultivation in up to four independently operating, oxygen-controlled microbioreactors. This is achieved by a feedback loop, which adjusts the perfusion velocity to keep the oxygen concentration at a preset level in each bioreactor individually. Furthermore, an implemented automated cell seeding procedure facilitates to distribute cells evenly in a stable scaffold before cultivation. The microbioreactor system can contribute to systematic investigations of crucial cultivation parameters in an oxygen-controlled environment and to more reproducible cultivation processes in tissue engineering.

## Introduction

The production of synthetic tissue constructs, which resemble native tissue, has great potential for regenerative medicine.^1^ For the cultivation of cells in a three-dimensional (3D) environment, bioreactor systems are mandatory to receive tissue-engineered grafts with uniform cell distribution, growth, and viability in a reproducible way.^2–5^ The application of bioreactor systems allows for the improvement of tissue quality by coping with limitations of static cultivation and by providing proper cultivation conditions for instance to mimic an *in vivo*-like environment.^3,6^

Several dynamic cultivation systems^7–11^ have been developed to overcome the limitations of static cultivation of 3D cell cultures, which are foremost linked to poor mass transfer. In static conditions, the distribution of nutrients and oxygen as well as the removal of waste products is solely dependent on diffusion, and thus limited to distances ∼100–200 μm.^12,13^ Using a microbioreactor, the supply with oxygen and nutrients can be significantly improved, especially when large tissue-engineered grafts are cultivated,^14–16^ with perfusion bioreactors having the highest potential to mitigate diffusional limitations.^17–19^

One of the most crucial cultivation parameters, which has to be considered for 3D cell culture, is the oxygen concentration (OC). Since every cell type demands a different OC for optimal cell growth and differentiation,^12,20,21^ the ability to ascertain optimal conditions is decisive for successful tissue engineering. Furthermore, the interpretation of oxygen data can provide information about cell growth and metabolic behavior in real time.^22–24^ To maintain OCs at an optimal level during a cultivation process, not only real-time sensing but also an automated feedback mechanism is needed.^3,6,25^ Nonetheless, many bioreactor systems lack integrated oxygen sensor technology^9–11^ or use sensor signals solely to observe present culture conditions.^26–28^

Besides integrated measurement instrumentation, the parallelization of bioreactors improves the functionality of a system significantly; not only because parallel experimental setups are much more time effective than subsequent approaches but also because the conduction of several parallel experiments allows for the investigation of several parameters in one run.^3,6,29^ Most bioreactor systems include the option of multiplexing.^9,10,30–32^ However, to display variations in an oxygen-controlled environment, bioreactors within a system have to be operating independently. Whereas the independent operation of parallel running bioreactors is described for mechanical stimuli,^33^ no such design is available for oxygen-controlled 3D cell culture.

If stable scaffolds are used for 3D cell culture, bioreactors can facilitate the generation of tissue-engineered grafts by not only maintaining optimal conditions but also by enabling uniform cell distributions during cell seeding before cultivation. Dynamic cell seeding procedures—conducted by bioreactors in an automated way—seem to have the highest potential for resulting in even cell distributions throughout stable scaffolds.^34–36^ This has to be considered, since varying cell distributions are leading to spatial nutrient and oxygen differences within a 3D cell culture, resulting in inhomogeneous growth.^37^

All these cultivation systems aim at the production of artificial tissue of high quality. However, for the fast and reproducible investigation of optimal culture conditions, cultivation systems need measurement instrumentation, the ability to control cultivation parameters as well as parallel and independent-operating bioreactors.^3,6^ However, currently available bioreactor systems lack at least one of these features. In addition, poorly controllable factors such as homogeneous cell seeding into 3D scaffolds are often not considered in bioreactor designs, and thus impeding the generation of comparable results.

Consequently, we developed a perfusion microbioreactor system for the oxygen-controlled cultivation of scaffold-based 3D cell cultures. The system is parallelizable up to four bioreactors, which are operated independently. Integrated needle-type microsensors (NTHs) are used to measure the OC in the geometric center of the 3D cell culture. The oxygen signals are processed in an automated feedback control to maintain OCs at a preset level by adjusting the perfusion speed for each bioreactor separately. In addition, an automated cell seeding procedure was implemented to facilitate the reproducible generation of homogeneous tissue. The bioreactors were optimized concerning a minimized dead volume, bubble-free operation, and a homogeneous flow distribution. Bioreactors were manufactured using stereolithography (SLA) and fused deposition modeling (FDM) 3D printing technology to provide a flexible and fast production process.

## Materials and Methods

### Microbioreactor

The perfusion microbioreactor was designed with the help of computer-aided design (CAD; SolidWorks 2016) and fabricated using the 3D printing technologies FDM (Ultimaker 2+) and SLA (FormLabs Form 2). Poly-l-Lactic acid (Innofil3D) was used for FDM, and DentalSG resin (FormLabs) for SLA. Gasket and 3D cell culture housing (3D-CCH) were molded using two-component silicone (Smooth-On MoldStar 15). To offer connectivity through luer-lock, hollow needles were glued into inlet, outlet, and sensor ports. The used materials were selected based on biocompatibility and the ability to be sterilized by autoclaving.

**FIG. 1.**
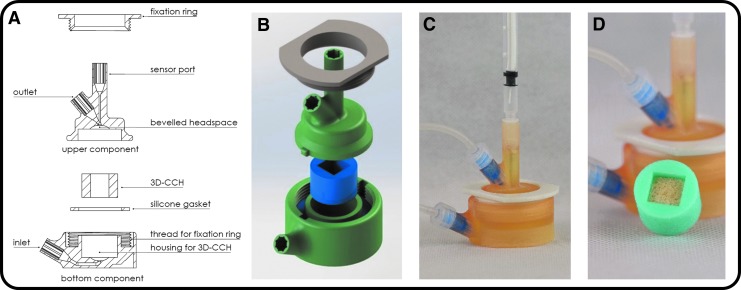
3D-printed microbioreactor for 3D cell culture. **(A)** Sectional drawing. **(B)** Explosion drawing. **(C)** Assembled with inlet/outlet/sensor. **(D)** Cubic bovine bone matrix scaffold in 3D-CCH. 3D, three dimensional; CCH, cell culture housing.

The microbioreactor design consists of five components (Fig. 1A). The bottom component contains the medium inlet and the housing for the 3D-CCH. The upper component contains the medium outlet and the sensor port. A silicone gasket between both components is used to seal the microbioreactor. The assembled components are kept in place using a threaded fixation ring, ensuring the long-term tightness of microbioreactors (Fig. 1B). The sensor port is designed to be used with syringe-guided NTHs allowing for measurements directly in the geometric center of the scaffold (Fig. 1C).

Due to the fabrication process through molding, the shape of the 3D-CCH can be varied and thus customized for different scaffolds. Consequently, scaffolds can be inserted press-fit in the microbioreactor—preventing leakage flow around the scaffolds—regardless of their shape. For the development of the microbioreactor, cubic scaffolds with edge lengths of 10 mm (as described in section “Cell culture”) were used (Fig. 1D).

Inlet and outlet are designed to avoid both dead spaces and entrapped air. The former is realized by opening up the inlet to the full diameter of the 3D-CCH, also resulting in a more uniform flow through the scaffold. The latter by a beveled head space in the upper component, which effectively transports air bubbles out of the microbioreactor.

### Parallelized microbioreactor system

**FIG. 2.**
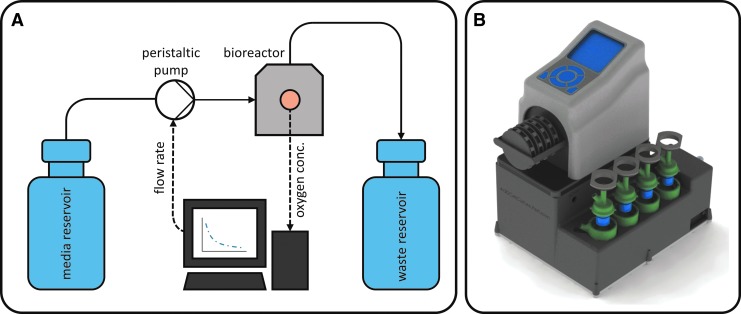
Parallelized microbioreactor system. **(A)** Process flow diagram of one bioreactor. Up to four microbioreactors (*gray*) can be used simultaneously. **(B)** Set up microbioreactor system with pump and four parallel microbioreactors.

The developed microbioreactor system consists of up to four microbioreactors, a peristaltic pump, and oxygen measurement instrumentation, which is assembled into one system (Fig. 2). In addition, software to display, log, and control the OC in each microbioreactor independently is provided (see section “OC control”). All bioreactors receive the cell culture medium from one medium reservoir. Used medium is collected in one waste reservoir. Each bioreactor is connected to the medium reservoir by gas-permeable tubes (PharMed Ismaprene; Ismatec), to allow for gas exchange with the surrounding atmosphere to keep O_2_ and CO_2_ concentrations in the medium at a constant level.

A peristaltic pump with low pulsation and four independently movable channels (Reglo ICC; Ismatec) is used for cell culture medium transport. For oxygen measurement, sensors are connected to electro-optical modules (EOMs) through ST/ST couplers (PreSens). To prevent false oxygen measurements due to temperature changes, the temperature of the cell culture medium is measured in the inlet of the bioreactor using a PT100 thermocouple (PreSens) in a flow-through cell. The PT100 is connected to the EOMs to be able to measure OCs while considering the current media temperature. RS232 serial port to USB adapters is used to connect EOMs and the pump to a PC. The system is set up in an incubator, which takes over the tasks of temperature control and setting up a CO_2_ atmosphere.

### Computation fluid dynamics

To show the microbioreactors' ability to provide a homogeneous flow through a 3D cell culture, a flow profile was simulated with and without an inserted scaffold by computational fluid dynamics (CFD) analysis using ANSYS CFX 17.0 through the ANSYS Workbench. Calculations were performed within a half model in the symmetric plane of the microbioreactor using a fine mesh (48,518 nodes, 123,979 elements) with tetrahedral structures for the main flow and prismatic elements for the sidewall areas.

A laminar incompressible flow with flow rates (FRs) between 10 and 250 μL/min was simulated with regard to impulse, mass, and energy conservation. The flow through inserted scaffolds was simulated using Darcy's law with scaffolds of 10 × 10 × 10 mm—according to scaffolds used for 3D cell culture in this study (see section “Cell culture”)—as a porous medium with pore sizes of 450 μm and 68% porosity (data provided by RTI Surgical for Tutobone scaffolds). The Darcian permeability (K) was estimated with K = 10^−9^ m^2^.^38–40^ Since the flow through the microbioreactor was found to be highly laminar in the considered range of FRs (Re <1), the quadratic loss coefficient was not taken into account for the simulations.

### Oxygen measurement

Needle-type oxygen microsensors (NTH-PSt1; PreSens) connected to EOMs (EOM-o2-micro; PreSens) were used for the oxygen measurements. A hollow needle of 0.4 mm diameter and 20 mm length was used to connect the NTH to the microbioreactor through luer-lock, to protect the optic fiber and to avoid deflection. Due to the bioreactors' design, the sensor tip of the mounted NTH is located directly in the geometric center of the 3D-CCH if the NTH is fully extended. A hole with a diameter of 0.5 and 5 mm length was drilled into the scaffold to ensure the correct positioning of the sensor tip in the 3D cell culture. OCs were measured every 120 s. According to the NTH-PSt1 manual, a two-point calibration was performed before each. Ambient air was used as the 21% oxygen reference and 100% CO_2_ as the 0% oxygen reference.

### OC control

Coding was performed with the visual programming language “G” using LabView 15 (National Instruments). The feedback mechanism allows for the independent control of OC by adjusting the FR of each channel of the peristaltic pump according to the oxygen sensor signal of the associated microbioreactor.

FRs between 10 and 250 μL/min were used for this approach. A proportional controller was coded to readjust the FR according to the oxygen signal. In accordance with the equation commonly used for proportional controllers, the proportional value (KP) was calculated by dividing the range of the regulating variable “FR” by the range of the control variable “OC.” The control deviation (e) was calculated by subtracting the OC-setpoint from the current OC:

\begin{align*}
F{R_{new}} = FR + KP \cdot e
\end{align*}

\[F{R_{new}}\left( {\frac{{\mu L}}{{min}}} \right) = FR\;\left( {\frac{{\mu L}}{{min}}} \right) + 11\;\left( {\frac{{min}}{{\mu L \cdot {\% _{{O_2}}}}}} \right){\text{ }} \cdot \left( {O{C_{current}} - O{C_{setpoint}}} \right).\]

To address the inertia of the system three checkpoints were integrated into the feedback loop to avoid high FR fluctuations. These comprise the control deviation, the trend of the OC during the last 10 iterations (ΔOC), and an OC-control time-out interval, which has to be defined before cultivation. After a FR adjustment the controller will remain inactive until the OC-control time-out interval elapsed. A FR adjustment is only possible if all the requirements listed in Table 1 are met. Otherwise, the FR will be kept constant.

**Table 1.** Oxygen Control, Criteria for Flow Rate Adjustments

*Possible adjustments*	*Control deviation*	*ΔOC*	*OC-control time-out interval*
Flow rate increase	*e* < 0	*ΔOC* < 0	Elapsed
Flow rate decrease	*e* < 0.5	*ΔOC* < 0	

OC, oxygen concentration.

The OC-control is embedded in an iterative loop (while loop), which is started after the initialization of the pump and the EOMs. For each iteration, data are requested from the EOMs, processed for the control, and (if necessary) written to the pump. In addition, a log-file is created to save the data hourly for further analysis. The following data are logged: cultivation time, OC, current flow rate, and whether the OC-control is active.

To demonstrate the functionality of the OC-control, oxygen levels were adjusted to 5%, 10%, and 15%, while one reactor remained uncontrolled.

### Cell culture

The human telomerase reverse transcriptase immortalized human mesenchymal cell (hMSC) line SCP-1^41^ modified to express green fluorescence protein (GFP) constitutively was used for the experiments. Cells were cultured at 37°C in a humidified 90% air and 10% CO_2_ atmosphere using Dulbecco's modified Eagle's medium (Merck) with 200 mM Glutamax (Gibco), 100 U/mL Penicillin/Streptomycin (Biochrom), and 10% fetal bovine serum (Biochrom). Decellularized cancellous bone matrix (Tutobone; RTI Surgical) of 10 × 10 × 10 mm was used as a scaffold for 3D cell culture. Perfusion was performed using a multichannel peristaltic pump (RegloICC; Ismatec). Flow velocities were adjusted between 10 and 250 μL/min to keep the OC in the 3D culture at a preset level. After the cultivation, cell nuclei were stained with 4′,6-diamidino-2-phenylindole (DAPI; Thermo Fisher). Cells were analyzed at 475 nm (GFP) and 461 nm (DAPI) using a Zeiss Axio Observer.Z1 microscope and the associated Zen software.

### Cell seeding

Cells were cultivated in T-flasks until a sufficient cell count was reached. Cells were trypsinized and counted using a hemocytometer (Brandt).

For static cell seeding, cells were resuspended in the medium to reach a concentration of 1 × 10^7^ cells/mL. The scaffolds were centrifuged (500 *g*, 5 min, room temperature) in cell culture medium to remove air bubbles. Subsequently, 250 μL of the cell suspension was seeded on the scaffolds as described elsewhere.^14^ After 1 h static incubation, scaffolds were placed into the bioreactors for dynamic cultivation.

For dynamic cell seeding, the loading volume had to be increased due to pumpability. To keep the number of seeded cells equal, cells were resuspended in a medium at a lower concentration of 1.25 × 10^6^ cells/mL. The scaffolds were placed into the microbioreactors and flushed with cell culture medium to remove air bubbles.

Subsequently, 2 mL of the cell suspension was seeded on the scaffolds using an oscillating flow at 1 mL/min. Oscillation was performed by changing the flow direction after every 200 μL inward and 100 μL outward directed flow. After reaching the total load volume of 2 mL, cells were cultivated statically for 1 h to allow the cells to adhere to the scaffold. Subsequently, dynamic cultivation was started. The dynamic cell seeding procedure was implemented in the bioreactor systems software and conducted automatically before cultivation, and thus referred to as “automated cell seeding.”

To evaluate whether OC and FR data of parallel runs can give information about the uniformity of cell distribution on the scaffolds, mean values and relative standard deviations of OCs and FRs were calculated every 5 h.

### Sterilization

The sterilization of thermostable materials was performed by autoclaving. For thermoplastic materials and oxygen sensors, a peroxide-based methodology was established, using 20% EtOH and 0.1% peroxyacetic acid for 45 min. Subsequently, the material was rinsed with demineralized water (3 × for 1 h) to remove residual peroxide and EtOH.^42,43^

## Results

### Microbioreactor and parallelized microbioreactor system

The bioreactors were manufactured using SLA and FDM. Gaskets and 3D-CHH were molded using two-component silicone. Rapid prototyping technologies offered a highly flexible design. The sterilization protocol and the functional sealing mechanism prevented contaminations reliably.

The microbioreactor system allows for the measurement and control of oxygen in up to four independent-operating microbioreactors. In combination with the implemented cell seeding procedure, a highly automated cultivation process could be performed. Due to the compact size of the system, it is suitable to fit in a bench-top incubator (for temperature control and setting up a CO_2_ atmosphere).

### Computation fluid dynamics

A CFD analysis with and without scaffold was done for various flow conditions. The calculated CFD model was used to investigate whether the designed microbioreactor is able to provide a homogeneous flow profile through an inserted 3D cell culture using FRs between 10 and 250 μL/min.

**FIG. 3.**
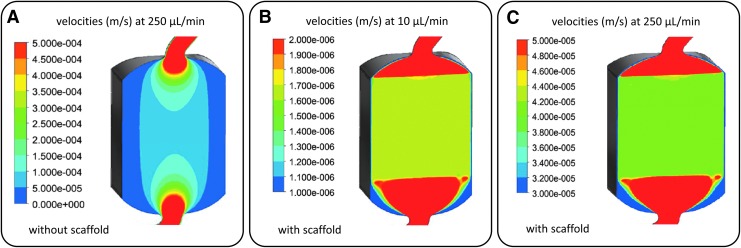
Computation fluid dynamics analysis of flow distributions in the developed microbioreactor. **(A)** Flow distribution and velocities at 250 μL/min without inserted scaffold. **(B)** Flow distribution and velocities at 10 μL/min. **(C)** Flow distribution and velocities at 250 μL/min.

At 250 μL/min, the flow profile of an empty microbioreactor shows high inhomogeneity with higher flow velocities in the center and areas with velocities close to 0 in the outer areas (Fig. 3A). The positive effect on the distribution of the media in the microbioreactor by opening the inlet to the full diameter of the 3D-CCH is already slightly visible though. If a scaffold is added to the model, a very homogeneous flow profile can be observed at 250 μL/min (Fig. 3C) as well as at 10 μL/min (Fig. 3B). At the given FRs, the observed flow velocities in the inserted scaffolds vary between 4 × 10^−4^ m/s at 250 μL/min and 1.6 × 10^−6^ m/s at 10 μL/min, respectively.

### OC control

To demonstrate the systems' capability of controlling the OC in a reliable and reproducible way, two OC-controlled runs were conducted. For each run, 2.5 × 10^6^ SCP-1 cells were seeded on Tutobone scaffolds statically and subsequently cultivated in four parallel microbioreactors. During the cultivation, oxygen levels of three microbioreactors were adjusted to 5%, 10%, and 15%, respectively, using the LabView-coded OC-control. The fourth microbioreactor remained uncontrolled.

**FIG. 4.**
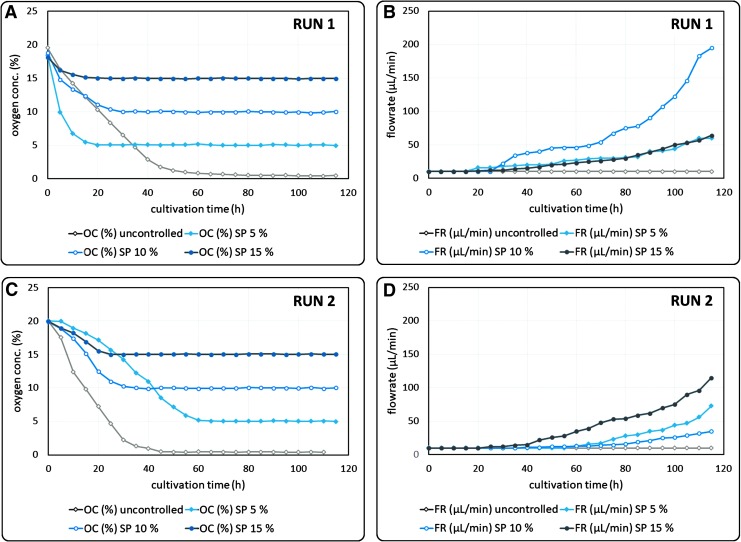
Cultivation of SCP-1 cells on Tutobone scaffolds, four parallel bioreactors, OC-control at different levels. OC and FR progressions of two runs of four parallel bioreactors, controlled at different oxygen levels (uncontrolled, 5%, 10%, and 15%). **(A)** OCs of Run 1. **(B)** FRs of Run 1. **(C)** OCs of Run 2. **(D)** FRs of Run 2. OC, oxygen concentration; FR, flow rate.

Before reaching the OC-setpoint, the FR was kept constant at the minimal FR of 10 μL/min. Once the OC fell below the OC-setpoint, the OC-control was triggered. Subsequently, the FR was adjusted according to the OC signal, to keep OCs at a preset level. Before OC-control activation, the oxygen consumption of the cells caused a decrease of OC levels in the center of the scaffolds. After OC-control activation, the controller was capable of adequately adjusting the FR to keep the OC constant during the whole cultivation period (Fig. 4A, C). An OC-time-out interval (see “OC control”) of 20 min was found to be suitable to minimize overshoot. As a result, the OC constantly remained in the range of “OC-setpoint ±0.5%” over a period of several days without great fluctuations. If OC levels were uncontrolled during the cultivation, OC levels dropped to anoxic conditions at ∼0% (Fig. 4A, C).

When comparing OC (prior OC-control) and FR progressions, it is noticeable that the slopes differ significantly in both runs (Fig. 4). This is most likely a consequence of the static cell seeding procedure, which was used for this approach. Both uneven cell distribution and different cell seeding efficiencies could be the reason for the differences in the observed OC and FR slopes. Consequently, an automated cell seeding procedure was developed to aim for higher homogeneity and thus reproducibility.

### Automated cell seeding

An automated (dynamic) cell seeding protocol was developed to distribute cells uniformly on a stable scaffold inserted into the developed microbioreactor. To demonstrate the functionality of the cell seeding protocol, SCP-1 cells were seeded on Tutobone scaffolds using different methods; first, the developed automated protocol and second, a static cell seeding approach. For each approach, three scaffolds were seeded with 2.5 × 10^6^ cells and subsequently cultivated in the developed microbioreactor system for 5 days. OCs were controlled at 15% O_2_. The progression of the OC and FR data was analyzed to evaluate the reproducibility of the developed automated and the static cell seeding procedure. After the cultivation, scaffolds were cut in half. Subsequently, the cell distribution was evaluated using fluorescence microscopy.

**FIG. 5.**
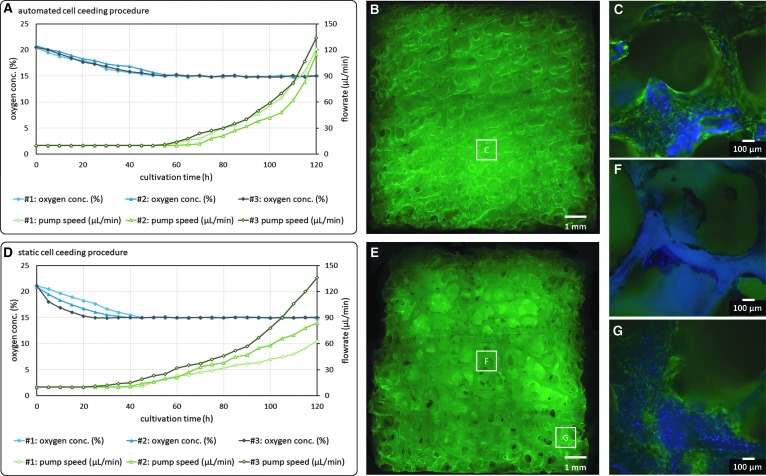
Cultivation of SCP-1 cells on Tutobone scaffolds, three parallel bioreactors, automated versus static cell seeding. OC- and FR progressions and evaluation of homogeneity. **(A)** Progression of OCs and FRs for an automated cell seeding approach (*n* = 3). **(B)** Homogeneous cell distribution after 120 h cultivation after automated cell seeding (GFP, 2.5 × magnification). **(C)** Automated cell seeding, overgrown scaffold section (GFP, DAPI, 10 × magnification). **(D)** Progression of OCs and FRs for a static cell seeding approach (*n* = 3). **(E)** Inhomogeneous cell distribution after 120 h cultivation after static cell seeding (GFP, 2.5 × magnification). **(F)** Static cell seeding, cell-free scaffold section (GFP, DAPI, 10 × magnification). **(G)** Static cell seeding, overgrown scaffold section (GFP, DAPI, 10 × magnification). GFP, green fluorescent protein; DAPI, 4′,6-diamidino-2-phenylindole.

The capability of the developed automated cell seeding protocol is clearly visible both in the OC and FR signals, and in the evaluated 3D cell cultures. The automated protocol resulted in a homogeneous cell distribution within the whole scaffold (Fig. 5B, C), whereas several cell-free spots could be observed when cells were seeded statically (Fig. 5E–G). Statically seeded cultures showed relative standard deviations up to 8.8% for OC slopes before OC-control (0–30 h) and up to 40% for FRs (30–120 h) (Fig. 5D). In contrast, relative standard deviations for OC slopes and FRs were mostly <2% (OCs) and 20% (FRs), respectively, when the developed automated cell seeding procedure was used (Fig. 5A).

## Discussion

### Microbioreactor and parallelized microbioreactor system

To facilitate the investigation of scaffold-based 3D cell cultures, we developed a microbioreactor system, which includes three important features to improve reproducibility: OC measurement and control, an automated cell seeding procedure, and parallelization. In this study, we assessed whether the implementation of these features in one cultivation system enables the generation of homogeneous 3D cell cultures in a reproducible way. In contrast to commercially available^31,32,35^ and tailor-made (micro)bioreactor systems,^9,10,25,30,44^ the developed microbioreactor system is able to monitor and control one of the most critical cultivation parameters, the OC, while offering the possibility of multiplexing (up to four bioreactors). In combination with the implemented automated cell seeding system, the developed microbioreactor system allows for fast and reproducible investigation of 3D cell cultures, which is a considerable need for TE.^3,6^

For the manufacture of the microbioreactors, rapid prototyping technologies such as SLA and FDM were used. The use of SLA and FDM allows for the fast production of microbioreactors and flexibility toward modifications for specific needs (e.g., different scaffold geometries). With the rapid advance of 3D printing technology and the general availability of 3D printers and materials, the fast production of smaller batches could make the system easily accessible for research facilities. Thus, the developed microbioreactor system represents a flexible and cost-efficient tool for the optimization and investigation of 3D cell cultures.

### Computation fluid dynamics

The use of a perfused bioreactor allows for an optimal supply with nutrients, while toxic metabolites are effectively removed from the cell culture.^4,17,18^ However, this is only the case if the flow is evenly distributed throughout the scaffold. Consequently, we investigated whether the developed microbioreactor is capable of providing a homogeneous flow profile throughout an inserted 3D cell culture by CFD.

It was found that the pressure drop over the scaffold is sufficient to distribute fluids uniformly throughout the 3D cell culture. When compared with other microbioreactor systems, which are already used for tissue-engineering approaches,^45^ the flow profile of the developed microbioreactor shows comparable characteristics. Consequently, the developed microbioreactor is capable of supplying even relatively large scaffolds/3D cell cultures with nutrients and oxygen in a homogeneous way.

### OC control

OC measurement instrumentation was integrated into the developed microbioreactor. Furthermore, a functional OC-control was developed in LabView 15. The control was able to keep the OC at a preset level with high accuracy (setpoint ±0.5%) and was able to reduce oxygen gradients in the 3D cell culture, which would result in inhomogeneous tissue quality.^14,46^ The proliferation potential of hMSCs, such as SCP-1 cells, is known to be unaffected by the present OC as long as the cells are cultivated at normoxic or hypoxic conditions. Some findings indicate even higher proliferation rates at hypoxic conditions, whereas the lack of oxygen at anoxic conditions leads to cell death of hMSCs and hence inhomogeneous 3D cell cultures.^12,25,47,48^

In accordance with these findings, the viability of the 3D cell cultures was found to be comparable when the OC was adjusted to 5%, 10%, or 15%, respectively, while the effect of the anoxic conditions on the cells in uncontrolled 3D cell culture resulted in high amounts of dead cells (data not shown). Regarding the progress of the FRs (Fig. 4B, D), their exponential shape suggests exponential cell growth, but apparently the slopes differ significantly. Since cell growth of SCP-1 cells is not affected by the present OCs at normoxic or hypoxic conditions,^47^ other effects have to be responsible for the differently increasing FRs at 15%, 10%, or 5%, respectively.

Since the number of cells, which were used for cell seeding, was equal, the different slopes are most likely a result of inhomogeneous cell distributions on the scaffold and different cell seeding efficiencies. Uneven cell distributions are most likely responsible for the differences in which the OCs drop before OC-control activation, too (Fig. 4A, C). The amount of metabolic active cells on a scaffold can be directly linked to the oxygen uptake rate (OUR) within a 3D cell culture.^49^ The measurement by a needle-type oxygen sensor solely displays the amount of oxygen-consuming cells surrounding the sensors, which offers the opportunity to compare cell distributions by comparing OUR signals of equally treated scaffolds.

Inhomogeneity is also noticeable by analyzing OC and FR progressions, for example, in the 3D cell culture, which was adjusted to 5% in run 1. Whereas the rapid OC decrease indicates a high amount of growing cells in the scaffold, the slow increase of the FR after OC-control activation is rather an evidence for a lower cell count. It is most likely that a high amount of cells had adhered in the surrounding of the oxygen probe, while significant areas of the scaffold remained cell free after cell seeding.

This leads to the conclusion that a functional OC-control is able to reduce oxygen gradients to maintain high cell viabilities in the 3D cell culture, but the OC-control alone is not sufficient to guarantee for homogeneous tissue quality. This is especially the case for 3D cell cultures with uneven cell distributions after cell seeding. Besides OC-control, a protocol for homogeneous cell seeding is hence mandatory for the generation of tissue of high quality in a reproducible way.

The OC-control allows for many different applications. First of all, it is possible to avoid the OC to drop below levels that would impede the viability of the 3D cell culture due to lack of oxygen. For the cell line used in this study (SCP-1), OCs <1% should be avoided to obtain a 3D cell culture of high viability. Critical OCs for optimal cell growth or cell differentiation could be easily determined by adjusting the OC to different levels in each bioreactor, and subsequently analyzing the viabilities of cultures or degree of differentiation.

The OC-control is implemented by a feedback loop, which adjusts the perfusion speed according to the OC in the associated bioreactor. One drawback of that mechanism is the variation of shear forces, which are applied to the cells. This is due to the variation in the FR during the cultivation process. This factor was not considered in this study. Nevertheless, since shear stress is known to affect the differentiation potential of hMSCs^45,50,51^ and is well considered in recent bioreactor studies,^45,51^ the decoupling of OC-control and FR is recently under investigation. This would make it possible to investigate whether the control of OCs could be combined with the control of shear forces in the developed microbioreactor system.

Furthermore, the OC has to drop from the initial to the preset OC before the control is activated, which requires a high amount of oxygen-consuming cells on the scaffold. This issue is highly linked to the pore size of the used scaffold. Large pore sizes and high porosities facilitate the oxygen supply of growing cells. Furthermore, large pores result in a smaller total surface area, and thus a lower amount of oxygen-consuming cells on an overgrown scaffold.^52^ Consequently, it is possible that the OC will not drop to the desired level, if scaffolds with large pore sizes are used, which naturally impedes the functionality of the OC-control.^25^

### Automated cell seeding

An automated cell seeding procedure was developed to facilitate uniform cell seeding in solid scaffolds. The use of a dynamic cell seeding procedure was shown to be much more efficient compared with static cell seeding approaches.^35,34^ These findings were confirmed in this work (Fig. 5). The concept of an oscillating flow profile, which was applied in other studies,^35^ was suitable to distribute cells throughout the whole scaffold homogeneously, with no visible cell-free spots.

However, even though the cell number used for the different protocols was equal, it must be considered that the seeding volume for the automated cell seeding had to be increased due to pumpability. The potential effect of different seeding volumes on the initial cell distribution was not further investigated though, since the main focus of the study was to implement the automated cell seeding protocol, and to investigate if unequal cell distributions can be observed in OC and FR progressions.

In addition, cell distribution is dependent on the pore size and geometry of the used scaffold. Consequently, the automated cell seeding procedure has to be optimized for different scaffolds before application. The integrated OC sensors and the OC-control of the developed system can be used for these optimization procedures efficiently. It was shown that cell seeding homogeneity is directly observable in OC and FR signals, since it is most likely that uneven cell distributions are the reason for differing OC- and FR progressions in parallel runs (Fig. 5A, D). Consequently, the developed system is also usable for the optimization and validation of dynamic cell seeding protocols.

## Conclusions

The developed microbioreactor system offers a novel option for the investigation of 3D cell cultures on solid scaffolds. The system is parallelizable up to four microbioreactors, whereas each microbioreactor can be adjusted to a preset OC individually. This is achieved by integrated OC measurement instrumentation and a developed OC-control, which adjusts the perfusion rate according to the present OC in an automated feedback loop. The OC-controlled cultivation of 3D cell cultures prevents OCs to drop below levels that could impede the viability of the cells. In addition, it enables optimization of cell growth and cell differentiation as a function of OC.

The implementation of an automated cell seeding system in combination with the functional OC-control and the parallelized setup facilitates the investigation and generation of tissue-engineered constructs with high homogeneity and viability in a reproducible way.

## References

[1] Drosse I., Volkmer E., Capanna R., De Biase P., Mutschler W., and Schieker M. Tissue engineering for bone defect healing: an update on a multi-component approach. Injury 39, S9, 2008 18804579 10.1016/S0020-1383(08)70011-1

[2] Carvalho J.L., de Carvalho P.H., and de Goes A.M. Advances in biomaterials science and biomaterials applications. In: Pignatello R., ed. Innovative Strategies for Tissue Engineering. London: InTECH Open Limited, 2013, pp. 295–313

[3] Ravichandran A., Liu Y., and Teoh S.H. Review: bioreactor design toward generation of relevant engineered tissues: focus on clinical translation. J Tissue Eng Regen Med 12, e7, 2018 28374578 10.1002/term.2270

[4] Kasper G., van Griensven M., and Pörtner R. Bioreactor Systems for Tissue Engineering. Berlin, Germany: Springer, 2009

[5] Amini A.R., Laurencin C.T., and Nukavarapu S.P. Bone tissue engineering: recent advances and challenges. Crit Rev Biomed Eng 40, 363, 2012 23339648 10.1615/critrevbiomedeng.v40.i5.10 PMC3766369

[6] Hansmann J., Groeber F., Kahlig A., Kleinhans C., and Walles H. Bioreactor in tissue engineering - principles, applications and commercial constraints. Biotechnol J 8, 298, 2013 23161827 10.1002/biot.201200162

[7] Sikavitsas V.I., Bancroft G.N., and Mikos A.G. Formation of three-dimensional cell/polymer constructs for bone tissue engineering in a spinner flask and a rotating wall vessel bioreactor. J Biomed Mater Res 62, 136, 2002 12124795 10.1002/jbm.10150

[8] Nishi M., Matsumoto R., Dong J., and Uemura T. Engineered bone tissue associated with vascularization utilizing a rotating wall vessel bioreactor. J Biomed Mater Res A 101, 421, 2013 22865391 10.1002/jbm.a.34340

[9] Bouet G., Cruel M., Laurent C., Vico L., Malaval L., and Marchat D. Validation of an in vitro 3D bone culture model with perfused and mechanically stressed ceramic scaffold. Eur Cell Mater 29, 250, 2009 10.22203/ecm.v029a19 25978114

[10] Piola M., Soncini M., Cantini M., Sadr N., Ferrario G., and Fiore G.B. Design and functional testing of a multichamber perfusion platform for tree-dimensional scaffolds. ScientificWorldJournal 2013**,** Article ID 123974, 2013 10.1155/2013/123974 PMC3885203 24453787

[11] Timmins N.E., Scherberich A., Früh J.A., Heberer M., Martin I., and Jakob M. Three-dimensional cell culture and tissue engineering in a T-CUP (tissue culture under perfusion). Tissue Eng 13, 2021, 2007 17590148 10.1089/ten.2006.0158

[12] Volkmer E., Kallukalam B.C., Maertz J., *et al.* Hypoxia preconditioning of human mesenchymal stem cells overcomes hypoxia-induced inhibition of osteogenic differentiation. Tissue Eng Part A 16, 153, 2010 19642854 10.1089/ten.TEA.2009.0021

[13] Lovett M., Lee K., Edwards A., and Kaplan D.L. Vascularization strategies for tissue engineering. Tissue Eng Part B Rev 15, 353, 2009 19496677 10.1089/ten.teb.2009.0085 PMC2817665

[14] Volkmer E., Drosse I., Otto S., *et al.* Hypoxia in static and dynamic 3D culture systems for tissue engineering of bone. Tissue Eng Part A 14, 1331, 2008 18601588 10.1089/ten.tea.2007.0231

[15] Carrier R.L., Rupnick M., Langer R., Schoen F.J., Freed L.E., and Vunjak-Novakovic G. Effects of oxygen on engineered cardiac muscle. Biotechnol Bioeng 78, 617, 2002 11992527 10.1002/bit.10245

[16] Rouwkema J., Rivron N.C., and van Blitterswijk C.A. Vascularization in tissue engineering. Trends Biotechnol 26, 434, 2008 18585808 10.1016/j.tibtech.2008.04.009

[17] Pörtner R., Nagel-Heyer S., Goepfert C., Adamietz P., and Meenen N.M. Bioreactor design for tissue engineering. J Biosci Bioeng 100, 235, 2005 16243271 10.1263/jbb.100.235

[18] Bancroft G.N., Sikavitsas V.I., and Mikos A.G. Design of a flow perfusion bioreactor system for bone tissue-engineering applications. Tissue Eng 9, 549, 2003 12857422 10.1089/107632703322066723

[19] Gaspar D.A., Gomide V., and Monteiro F.J. The role of perfusion bioreactors in bone tissue engineering. Biomatter 2, 167, 2012 23507883 10.4161/biom.22170 PMC3568103

[20] Fehrer C., Brunauer R., Laschober G., *et al.* Reduced oxygen tension attenuates differentiation capacity of human mesenchymal stem cells and prolongs their lifespan. Aging Cell 6, 745, 2007 17925003 10.1111/j.1474-9726.2007.00336.x

[21] Holzwarth C., Vaegler M., Gieseke F., *et al.* Low physiologic oxygen tension reduce proliferation and differentiation of human multipotent mesenchymal stromal cells. BMC Cell Biol 11, 11, 2010 20109207 10.1186/1471-2121-11-11 PMC2827377

[22] Super A., Jaccard N., Cardoso Marques M.P.C., *et al.* Real-time monitoring of specific oxygen uptake rates of embryonic stem cells in a microfluidic cell culture device. Biotechnol J 11, 1179, 2016 27214658 10.1002/biot.201500479 PMC5103178

[23] Weyand B., Nöhre M., Schmälzlin E., *et al.* Noninvasive oxygen monitoring in three-dimensional tissue cultures under static and dynamic culture conditions. Biores Open Access 4, 266, 2015 26309802 10.1089/biores.2015.0004 PMC4497672

[24] Streeter I., and Cheema U. Oxygen consumtion rate of cells in 3D culture: the use of experiment and simulation to measure kinetic parameters and optimise culture conditions. Analyst 136, 4013, 2011 21804989 10.1039/c1an15249a

[25] Volkmer E., Otto S., Polzer H., *et al.* Overcoming hypoxia in 3D culture systems for tissue engineering of bone in vitro using an automated, oxygen-triggered feedback loop. J Mater Sci Mater Med 23, 2793, 2012 22843167 10.1007/s10856-012-4725-0

[26] Lee P.S., Eckert H., Hess R., *et al.* Developing a customized perfusion bioreactor prototype with controlled positional variability in oxygen partial pressure for bone and cartilage tissue engineering. Tissue Eng Part C Methods 23, 286, 2017 28401793 10.1089/ten.TEC.2016.0244

[27] Chan W.Y., and Chong C.K. Perfusion bioreactors improve oxygen transport and cell distribution in esophageal smooth muscle construct. IFMBE Proceedings. Berlin, Germany: Springer, 2009, p. 23

[28] Janssen F.W., Oostra J., van Oorschot Av, and van Blitterswijk C.A. A perfusion bioreactor system capable of producing clinically relevant volumes of tissue-engineered bone: in vivo bone formation showing proof of concept. Biomaterials 27, 315, 2006 16125223 10.1016/j.biomaterials.2005.07.044

[29] Meyvatson I., and Beebe D.J. Cell culture models in microfluidic systems. Annu Rev Anal Chem 1, 423, 2008 10.1146/annurev.anchem.1.031207.113042 20636085

[30] Sailon A.M., Allori A.C., Davidson E.H., Reformat D.D., Allen R.J., and Warren S.M. A novel flow-perfusion bioreactor supports 3D dynamic cell culture. J Biomed Biotechnol 2009, Article ID: 873816, 2009 10.1155/2009/873816 PMC2796393 20037739

[31] 3D Biotek. 3D Biotec perfusion bioreactor. [Online]. Available at: http://3dbiotek.com/Documents/Bioreactor_Brochure.pdf (Accessed 57, 2018)

[32] SKE Research Equiptment. SKE inflow perfusion bioreactor. [Online]. Available at:http://ske.it/products/inflow-modular-perfusion-bioreactor (Accessed 57, 2018)

[33] Brady M.A., Vaze R., Amin H.D., Overby D.R., and Ethier C.R. The design and development of a high-throughput magneto-mechanostimulation device for cartilage tissue engineering. Tissue Eng Part C Methods 20, 149, 2014 23721097 10.1089/ten.tec.2013.0225 PMC3910453

[34] van den Dolder J.V.D., Spauwen P.H.M., and Jansen J.A. Evaluation of various seeding techniques for culturing osteogenic cells on titanium fiber mesh. Tissue Eng 9, 315, 2003 12740094 10.1089/107632703764664783

[35] Wendt D., Marsano A., Jakob M., Heberer M., and Martin I. Oscillating perfusion of cell suspensions through three-dimensional scaffolds enhances cell seeding efficiency and uniformity. Biotechnol Bioeng 84, 205, 2003 12966577 10.1002/bit.10759

[36] Koch M.A., Vrij E.J., Engel E., Planell J.A., and Lacroix D. Perfusion cell seeding on large porous PLA/calcium phosphate composite scaffolds in a perfusion bioreactor system under varying perfusion parameters. J Biomed Mater Res A 95, 1011, 2010 20872752 10.1002/jbm.a.32927

[37] Galban C.J., and Locke B.R. Effects of spatial variation of cells and nutrient and product concentrations coupled with product inhibition on cell growth in a polymer scaffold. Biotechnol Bioeng 64, 633, 1999 10417211

[38] Innocentini M.D.M., Faleiros R.K., Pisani R., Thijs I., Luyten J., and Mullens S. Permeability of porous gelcast scaffolds for bone tissue engineering. J Porous Mater 17, 615, 2010

[39] Truscello S., Kerckhofs G., Van Bael S., Pyka G., Schrooten J., and Van Oosterwyck H. Prediction of permeability of regular scaffolds for skeletal tissue engineering: a combined computational and experimental study. Acta Biomater 8, 1648, 2012 22210520 10.1016/j.actbio.2011.12.021

[40] Sanz-Herrera J.A., Kasper C., van Griensven M., Garcia-Aznar J.M., Ochoa I., and Doblare M. Mechanical and flow characterization of sponceram carriers: evaluation by homogenization theory and experimental validation. J Biomed Mater Res B Appl Biomater 87, 42, 2008 18395821 10.1002/jbm.b.31065

[41] Böcker W., Yin Z., Drosse I., *et al.* Introducing a single-cell-derived human mesenchymal stem cell line expressing hTERT after lentiviral gene transfer. J Cell Mol Med 12, 1347, 2008 18318690 10.1111/j.1582-4934.2008.00299.x PMC3865677

[42] Shearer H., Ellis M.J., Perera S.P., and Chaudhuri J.B. Effects of common sterilization methods on the structure and properties of poly(d,l lactic-co-glycolic acid) scaffolds. Tissue Eng 12, 2717, 2006 17518641 10.1089/ten.2006.12.2717

[43] Yoganarasimha S., Trahan W.R., Best A.M., *et al.* Peracetic acid: a pracitcal agent for sterilizing heat-labile polymeric tissue-engineering scaffolds. Tissue Eng Part C Methods 20, 714, 2014 24341350 10.1089/ten.tec.2013.0624 PMC4152794

[44] Bhaskar B., Owen R., Bahmaee H., Rao P.S., and Reilly G.C. Design and assessment of a dynamic perfusion bioreactor for large bone tissue engineering scaffolds. Appl Biochem Biotechnol 185, 555, 2018 29235057 10.1007/s12010-017-2671-5

[45] Egger D., Fischer M., Clementi A., Ribitsch V., Hansmann J., and Kasper C. Development and characterization of a parallelizable perfusion bioreactor for 3D cell culture. Bioengineering 4, 51, 2017 10.3390/bioengineering4020051 PMC5590478 28952530

[46] Malda J., Klein T.J., and Upton Z. The roles of hypoxia in the in vitro engineering of tissues. Tissue Eng 13, 2153, 2007 17516855 10.1089/ten.2006.0417

[47] Maertz J., Saller M., Kallakukalam B.C., Docheva D., Schieker M., and Volkmer E. Hypoxia and HIF-1alpha-regulation do not affect proliferation of human mesenchymal stem cells but inhibit osteogenic differentiation in-vitro. JSM Regen Med Bio Eng 3, 1019, 2015

[48] Carrancio S., López-Holgado N., Sánchez-Guijo F.M., *et al.* Optimization of mesenchymal stem cell expansion procedures by cell separation and culture conditions modification. Exp Hematol 36, 1014, 2008 18468767 10.1016/j.exphem.2008.03.012

[49] Lambrechts T., Papantoniou I., Sonnaert M., Schrooten J., and Aerts J.M. Model-based cell number quantification using online single-oxygen sensor data for tissue engineered perfusion bioreactors. Biotechnol Bioeng 111, 1982, 2014 24771348 10.1002/bit.25274

[50] Chen H.C., and Hu Y.C. Bioreactors for tissue engineering. Biotechnol Lett 28, 1415, 2006 16955350 10.1007/s10529-006-9111-x

[51] Williams C., Kadri O.E., Voronov R.S., and Sikavitsas V.I. Time-dependent shear stress distributions during extended flow perfusion culture of bone tissue engineered constructs. Fluids 3, 25, 2018

[52] Loh Q.L., and Choong C. Three-dimensional scaffolds for tissue engineering applications: role of porosity and pore size. Tissue Eng B Rev 19, 485, 2013 10.1089/ten.teb.2012.0437 PMC3826579 23672709

